# English community pharmacists’ experiences of using electronic transmission of prescriptions: a qualitative study

**DOI:** 10.1186/1472-6963-13-435

**Published:** 2013-10-23

**Authors:** Sara Garfield, Ralph Hibberd, Nick Barber

**Affiliations:** 1 The Department of Practice and Policy UCL School of Pharmacy, Mezzanine Floor, BMA House, Tavistock Square, London, UK; 2 The Centre for Medication Safety and Service Quality, Imperial College Healthcare NHS Trust, London, UK

## Abstract

**Background:**

The Electronic Prescription Service Release 2 (EPS2) in England has been designed to provide electronic transmission of digitally-signed prescriptions between primary care providers, with the intent on removing the large amounts of paper currently exchanged. As part of a wider evaluation of the EPS service, we wished to explore pharmacists’ experience with the new system and their perceptions of its benefits and any associated problems.

**Methods:**

We conducted semi-structured telephone interviews with community pharmacists using EPS2. We used a purposive sampling technique to obtain views from pharmacists working in pharmacies with a range of sizes and locations and to include both independent pharmacies and multiples. Interviews were transcribed verbatim and coded using grounded theory to identify the main factors that have influenced deployment and implementation in the eyes of respondents. QSR Nvivo was used as to aid in this process.

**Results:**

It became apparent from the analysis that respondents perceived a wide range of advantages of EPS including improved safety, stock control, time management and improved relationships between pharmacy and General Practice staff. Respondents did not perceive a large difference in terms of work processes or development of their professional role. A large number of problems had been experienced in relation to both the technology itself and the way it was used by General Practices. It became apparent that work-around procedures had been developed for dealing with these issues but that not all these problems were perceived as having been addressed sufficiently at source. This sometimes had implications for the extent of EPS2 use and also limited some of the potential advantages of the EPS2 system, such as reduced effort in the management of prescription reimbursement. Respondents made suggestions for future improvements to EPS2. While interview data demonstrated that there were some feedback procedures in place, these were not regarded as being sufficient by the majority of respondents.

**Conclusions:**

Whilst pharmacists perceived a wide range of benefits of EPS, a large number of problems had been experienced. Despite these difficulties, no pharmacists expressed an overall negative view.

## Background

The Electronic Prescription Service (EPS), NHS England’s service for the electronic transmission of prescriptions, has been designed to support a change moving from paper based transmission of prescriptions in primary care to transmission in a digital format including a digital signature. First announced in 2003, and subsequently adopted in 2005 as one part of the National Programme for Information Technology, the establishment and management of EPS has been managed by Connecting for Health, an agency of the Department of Health.

The role of EPS is a fundamentally simple one: it allows the transmission of prescription messages and digitally-signed prescriptions via a central network and server infrastructure, the Spine, from where they can be downloaded by dispensing contractors including community pharmacists, dispensing appliance contractors and dispensing doctors. Prescriptions are then subsequently passed on electronically to NHS Prescription Services for reimbursement. There have been two releases of EPS (EPS1, EPS2). EPS1, in use since 2005, prints a barcode on the prescription form. This can be scanned by the pharmacy to initiate a download of data, although the paper prescription still remains the legal entity for dispensing and reimbursement. In EPS2 a digital prescription is sent to the Spine, which a pharmacy can then download and dispense. The patient can nominate a specific pharmacy and the prescription is directed there. Completion of dispensing by the community pharmacy initiates a claim message to be sent to NHS Prescription Services, the body responsible for calculating reimbursements and remunerations, obviating the need for them to receive paper prescriptions.

The introduction of EPS2 has been associated with a number of potential benefits for patients and staff. These include the reduction of the number of illegible prescriptions received, the promotion of increased use of repeat dispensing prescriptions which should promote better management of repeat prescriptions, and reduced effort in the management of prescription reimbursement [1-3]. However, as Cornford, Doukidis and Forster eloquently summarised, the outcomes from any workplace intervention emerge from the fit between the technology introduced and change to the work processes around the use of the technology itself and not simply staff interaction with technology [4].

Although there are many tools that exist for the evaluation of systems a priori, we cannot be certain as to how these systems will operate in the real world. Related work on the use of computerized physician order entry (electronic prescribing) and electronic transfer of prescriptions in other countries shows that unintended consequences can emerge from the introduction of electronic prescribing related services [5-7]. Consequently, there is a need to explore how such systems operate in the real-world and how errors in operation are mitigated and defended against.

Although the design of the EPS2 follows the traditional paper process, there are a number of changes which might lead to the adoption of new work practices. Firstly, the service involves the introduction of a new intermediary in the process of prescription transmission, the Spine. Prior to EPS, the transfer of the paper prescription from the GP practice to another responsible agency was transparent and could be audited. In the case of EPS whilst the process of transmission is potentially more reliable, there is an intermediary system, which represents the boundary of the GP practice’s responsibility but whose operation is not visible to either GP practice or community pharmacy except with regard to the delivery of the prescription. Indeed, in the case of community pharmacy, transmission of the prescription is not direct to the community pharmacy but via a server acting as a community pharmacy message handler. The hand-over from the Spine to the message handler is reported through a web portal, but the Spine does not hold any data on what happens to the prescription that the message handler does not send back. For example, if a prescription got stuck in the message handler, this would not be visible.

Secondly, the service does not cover all prescription items; some fall outside of the scope of the service, including controlled drugs and items that are not mapped to the Dictionary of Medicines and Devices (DM + D). In these cases, GP practice systems might offer the option of splitting the prescription between paper and electronic prescriptions, or simply require that the prescription is printed on paper. There are management implications for both of these scenarios. Where the prescription has been split, the community pharmacy needs to be aware that the patient’s prescription will be in two parts and that there is a need to reconcile these. In those cases where the prescription is provided on a paper document, the community pharmacy needs to be aware that even in those cases where the patient has a community pharmacy nomination that a paper prescription needs to be obtained.

Finally, the location at which repeat dispensing prescriptions are stored moves from the community pharmacy to the Spine. Each issue of a repeat dispensing prescription is issued according to a fixed schedule, which is either set explicitly by the prescriber, or is set by the Spine automatically. Each issue will be available to the nominated community pharmacy a week before the medication is required. However, the Spine only knows when the prescription is due if the community pharmacy puts a notification on the system that it has been dispensed. From feedback received, we understood that there might be delays in this process.

Undoubtedly there are further subtle changes that emerge from the operation of EPS which need to be taken into account, and which will require change in the manner community pharmacies manage prescriptions.

In this paper, we address five main research questions related to pharmacy staff interaction with the new service. First, how have work processes changed as a consequence of the introduction of EPS2? Second, what advantages do pharmacists perceive in the service and how have the goals that community pharmacy expects to fulfill changed? Third, what process disruptions emerge as a consequence of human or system error? Fourth, how do community pharmacies mitigate these process disruptions? Finally, what are the feedback procedures available to community pharmacy staff when they experience these disruptions?.

This paper follows on from some very early pilot work with community pharmacies in which the authors reported some preliminary findings but suggested further studies to establish how the system operated when more mature. We therefore conducted further interviews when the system was more mature and users were more settled into their experience.

## Methods

This study aimed to provide data that would support the interpretation of concurrent quantitative data focusing on dispensing accuracy and dispensing interventions [8]). It appeared most appropriate to conduct semi-structured interviews with participants as we were seeking pharmacists’ viewpoints, which cannot be obtained by observation. This provided an opportunity to confirm established knowledge about the service and also to explore the issues that participants identified as particularly salient to the research questions.

## Ethical requirements

This was part of a larger project which had been classed as a service evaluation and hence not requiring ethical committee approval. This status was confirmed with the chair of the Cambridgeshire I Research Ethics Committee.

Respondents were provided with an information sheet, which reminded them that participation was voluntary and of their right to withdraw at any time. Verbal consent was gained from respondents at the start of the interview. All data collected were anonymised in accord with the standard practices adopted by this project.

### Sampling

In this study our focus was on the community pharmacists who used EPS2 and who were responsible for the reconfiguration of their workplaces to support activity. Given the aim of the study to provide explanation of the results obtained from the study of dispensing reliability, the initial group of respondents was drawn from those community pharmacies already studied. However, these did not represent the only sites using EPS2 and consequently, we also recruited additional sites to provide further insight into the operation of EPS2 in community pharmacy. Data obtained from the NHS Business Authority showed that at the start of this research, in July 2012 there were 598 pharmacy contractors offering EPS2 services in the UK. We sampled from these, using a maximum variation purposive sampling technique in order to obtain views from pharmacists working in pharmacies with a wide range of size, in a range of geographical locations to include pharmacists working in both independent pharmacies and multiples and to include pharmacists with varying levels of experience with EPS2.

### Sample

This study was qualitative in nature. We had been following all the early adopter pharmacies since EPS2 went live. Although there were over 500 pharmacies that were offering the service at the time of our study, very few had a GP practice that was regularly sending prescriptions by EPS2; in our estimation there were about 78 pharmacies in England that were using the service sufficiently frequently to be able to comment on it with some authority, and we drew our sample from these. We conducted data analysis concurrently with the interviews and as such continued interviewing until theoretical saturation was reached. This occurred when 13 pharmacists had been interviewed.

### Recruitment

Researchers who were visiting the community pharmacies previously recruited to our wider study took letters with them asking them to participate in this specific part of the study. In addition, we asked one of the Primary Care Trusts (PCTs) which had deployed EPS2 widely to suggest some community pharmacies. These community pharmacies were sent an email followed by a telephone call. We asked pharmacists to either contact SG directly, or to indicate to the researcher they were happy to participate. All community pharmacies approached were in PCTs that were proactive in promoting EPS2.

### Data collection

Individuals agreeing to participate were contacted by telephone to arrange a mutually convenient time to conduct the interview. The interviews were conducted by telephone using a semi-structured interview schedule which addressed the research objectives (see Additional file 1). With verbal consent of the respondents, the interviews were audio recorded.

### Analysis

Interviews were transcribed verbatim and coded using the four stages of grounded theory [9] to identify the main factors that have influenced deployment and implementation in the eyes of respondents. QSR Nvivo 8 was used as to aid in this process. Themes that were pertinent to the research objectives were initially identified from a literature review and preliminary findings from pilot work. Interviews were then coded into these existing categories. New categories and sub categories were also developed throughout the process, allowing an index structure to be built. For example, some problems and concerns identified from the pilot work, such as problems with missing prescriptions, were part of the initial coding frame. However, other problems such as the large amount of printing were identified during the study and new codes were therefore created.

## Results

Sixteen pharmacists were approached and thirteen agreed to participate, giving a response rate of 81%. Of the thirteen interviewees, five worked in independent pharmacies, three in small multiples, four in chain pharmacies and one in a supermarket pharmacy. Nine of the pharmacies had only a single pharmacist working at any one time and the other four had two. The number of dispensers ranged from one to ten per pharmacy, with the mean being three.

The themes identified comprised: motivation for moving to an EPS2 system, training, types of prescriptions received via the EPS2 system, process change to support EPS2, overall views of EPS2, perceived advantages EPS2, perceived changes to professional role related to EPS2, problems associated with EPS2, suggested improvements to the EPS2 system, and feedback procedures available to pharmacists. Each of these are next described.

### Motivation for moving to an EPS2 system

The interview data demonstrated that respondents decided to sign up for EPS2 for a range of reasons. These included sanctioning by others: six of the thirteen respondents reported that the PCT was a motivating force, two that they had to gone live because the GP practice near them had done so, and two that the decision had been taken by their head office. Other reasons reported for going live included wanting to be at the forefront (four respondents), being of the opinion that it would be good for business (two respondents) and the perception that it would save time (one respondent).

‘The large surgery next door to us were very keen on doing a trial, and they sort of semi volunteered us for it’. [interview pharmacy 5].

‘We wanted to be at the forefront and influence the way that the things are implemented locally’. [interview pharmacy 12].

### Training

The majority of interviewees reported having had some training to help them implement EPS2. Nine of the thirteen respondents had received training from their software provider, two from Connecting for Health and one from the head office of their pharmacy chain. However, one respondent stated that he had not received any training. Five respondents were of the view that self learning had played an important role in the implementation of EPS2.

‘I think that we found most of the stuff out ourselves in the end’. [interview pharmacy 13].

Training was delivered in a variety of formats, sometimes in combination, including face to face (six respondents), on line training (three respondents), videos (two respondents) and the provision of written information (four respondents).

It became apparent from the analysis that whilst many respondents were generally positive about the training they had received, there was some room for improvement in some cases. Seven respondents reported that the training had been useful and sufficient. However, two were of the view that it had been quite brief and one was of the opinion that learning more trouble-shooting during training would be helpful. One of the respondents discussed the fact that although he had received good training, he was one of the first sites to go live and that others might not now receive training that was as comprehensive. The timing of training was also discussed by several respondents. Three respondents reported that the timing had been good. However, two were of the view that the training had been given too early, as there had then been delays going live, resulting in it being forgotten. Conversely, two respondents were of the view that it had been given too late, after they had already learnt how to use it themselves; one of these two respondents said that this was because of previous experience of the trainer that it had been given too early at other sites. One respondent had been given training both before and after going live and found that supportive. Another also suggested that this would be a helpful approach.

‘I think that a lot of people when it comes to training, they are very negative about pointing out negatives. …A lot of the problems that you have are annoying things and they can be sorted out if you know how to sort them out’. [interview pharmacy 8].

‘It was very good because basically it was logging on to the website and over time basically looking at it, and it was done before we went live as well, so we were ready for it’. [interview pharmacy 10].

‘It was due to go live and then it didn’t and then by the time it did it was a number of months and then of course you had forgotten what you were told’. [interview pharmacy 6].

‘In a way you need it before you start getting prescriptions as you need training around nominations and so forth, but I think that there will definitely be a place for a follow up, even just a short one - three or four weeks, having put on new prescriptions’. [interview pharmacy 8].

### Types of prescriptions received electronically

Eleven of the thirteen interviewees reported that they had started receiving and dispensing EPS2 prescriptions; they had received both acute and repeat prescriptions. The other two pharmacies (pharmacies 10 and 11) were EPS2-live but had not yet started receiving EPS2 prescriptions.

The majority of respondents (eight) also reported receiving electronic repeat dispensing prescriptions. Of these, three had not been involved with paper repeat dispensing and two had expanded their repeat dispensing service following the introduction of EPS2. In at least one area the expansion of the service was a result of new incentives for repeat dispensing from the PCT which coincided with the introduction of EPS2.

‘The repeat dispensing has increased a lot, however, I wouldn’t like you to think that it is because of this, because we have heard that the GPs have been recently incentivised to put patients on to repeat dispensing’. [interview pharmacy 14].

### Process change to support EPS2

The majority of respondents reported that there had been no or minimal process changes following the introduction of EPS2. However, one interviewee was of the opinion that it had simplified the process. Six respondents reported that new standard operating procedures had been written either by themselves or their head office. However, two had not created new standard operating procedures.

‘We are still having the dispensers, or the pre-reg or the technicians. We are still having to take the medicine off the shelf, and still having to put the labels on themselves, and if a green [paper] prescription comes in or an electronic prescription, the process is essentially the same’. [interview pharmacy 7].

### Overall views of EPS2

Five respondents expressed an overall view of EPS2. Two were positive and the other three were mixed.

‘Out of ten, I’d give it an eight if that makes sense. … On the whole it has been a positive process’. [interview pharmacy 7].

### Perceived advantages of EPS2

A range of benefits were reported to have been experienced by pharmacists using EPS2. Respondents were asked about the effect of EPS2 on safety. Ten were of the opinion that using the EPS2 system reduced errors, two did not think it made any difference and one did not express an opinion. Of those of the view that EPS2 improved safety, eight stated that labeling errors were reduced as information did not have to be manually transferred from the prescription. However, there was an acknowledgment by some of these respondents that accurate labeling still relied on the prescriber entering the correct information on the prescription in the first place. Three respondents also reported that reduced pressure from a more even workflow would (one respondent) or did (two respondents) help reduce errors.

‘Very straightforward and very accurate and very little chance of making dose errors, because it’s obviously just a duplicate of what the doctor has put on his machine, so if it is going to be an error of dose it comes from him’. [interview pharmacy 15].

The majority of respondents (eight) reported that EPS2 helped even out workflow and seven respondents were of the view that the service saved (six) or would save (one) time overall by reducing time spent on collecting prescriptions from the GP practice, labeling and/or processing end of month claims. However, one respondent was of the view that workflow had improved at the beginning but not any longer.

‘I suppose its … more of a steady flow because we might download 2 or 3 times a day and the surgery will do the same their end. Whereas before we would collect all the prescriptions once a day, so the repeat prescriptions. Now we can go down in the morning and if [we] have it quiet we can check if there are any more to do’. [interview pharmacy 6].

‘When you get the prescriptions electronically, with printing the labels, it has become…just work-wise it has become a lot quicker. Because, for example if you have an ordinary prescription you have to look for the item and find out what it is, enter the dose manually, but with this one it is done for you already [interview pharmacy 9].

When asked how EPS2 affected relationships between pharmacies and GP Practices, the majority of respondents (seven) were positive. Respondents reported that EPS2 encouraged communication and strengthened relationships due to shared problem solving. One respondent reported that the pharmacy had already had a good relationship with the clinical staff at the GP practice but this had now expanded to administrative staff as well. Another was of the view that EPS2 gave doctors a greater appreciation of the role pharmacists play in interpreting their prescriptions. Two others reported that it was now quicker to receive amended prescriptions where necessary and one that it reduced emergency supplies at the doctors’ request as the doctor could now send a valid prescription immediately. However, one respondent was of the opinion EPS2 had not affected pharmacist-GP relationships.

‘Well because we are working together with it, it is new to them and new to us, we kind of, you are on the same side aren’t you. We’re all trying to get to the same conclusion, but also it is, when we have a problem, and we are ringing up and they are dealing with it more efficiently than they were before’. [interview pharmacy 6].

‘I wouldn’t think that it has made em. much difference if any. It hasn’t because we still are the same as when we were dealing with paper really’. [interview pharmacy 1].

Views were split as to whether or not EPS2 helped to improve stock control and reduce medication owings. Five respondents reported that owings were reduced as the prescriptions came in earlier, giving the pharmacist time to order in stock required before the patient arrived. One respondent stated that he therefore needed to keep less items in stock. However, three respondents expressed the view that EPS2 had not reduced owings because they already had a good stock control system due to good patient–pharmacy communication when ordering repeats or because most owings were due to manufacturers being out of stock; a factor which EPS2 was unable to reduce.

‘Therefore even if there is something missing you have got a good chance that it will come in before the patient comes in. Whereas with the green [paper] prescription, the normal one it has to be done there and then, as it is usually brought in by hand, and the patient if there is anything missing you don’t have that extra time to get that item.’ [interview pharmacy 15].

‘Most of them, the manufacturers are out of stock now, which are pretty bad at the minute and I would not say that there has been much impact that way.’ [interview pharmacy 14].

A number of other benefits were identified by a smaller numbers of respondents. Benefits for pharmacists included taking a perceived step forward (two respondents), retaining access to prescriptions rather than needing to send the prescriptions off and therefore being able to deal with queries on past prescriptions more efficiently (one respondent), the potential that EPS2 would lead to pharmacist access to clinical records in the future (one respondent), the potential that administrative time dealing with repeat dispensing would be lower (one respondent) improved relationships with patients (one respondent) and high street pharmacies (pharmacies on the main shopping street) receiving more prescriptions (one respondent). Perceived benefits for patients included less waiting time in the pharmacy (two respondents), quicker processing of repeat requests (one respondent) and a more efficient repeat dispensing service (one respondent). Perceived benefits for the GP practice reported by individual pharmacists included saving time, saving paper and increased signing flexibility as the prescription was not tied down to one doctor or one location. A general benefit suggested by two respondents was that there were less prescriptions lost in the system. This is an important finding to consider in the context of considering missing electronic prescription as described in the problems and concerns section below.

‘Whereas before if you handed a repeat form in, it should have come to us, but we all know that prescriptions go. So there is a lot less wayward prescriptions’. [interview pharmacy 8].

### Changes to professional roles associated with EPS2

The majority (nine) of respondents expressed the view that EPS2 had not impacted on the professional role of pharmacists and that they were still carrying out the same roles as before. However, three respondents were of the view that improved workflow and safety of EPS2 had freed up some of their time from dispensing to allow them to carry out other professional roles. In addition, two respondents expressed the opinion that EPS2 had improved their professional relationship with patients and one respondent saw the improved relationship with the GP practice as an advancement of their professional role.

‘Professional, I don’t think that anything has changed, because we are still dispensing or seeing the patients and speaking to them, and so I don’t think that it has changed or made any changes with the professional role’. [interview pharmacy 9].

‘It frees up your time a lot more, and because, so as I say because you can organise things a lot better, you know when, you can choose when to check the prescriptions and you know, there is less urgency about it. … So you would be able to tie it in with other things like MURs [medication use reviews] and other services, and you know, that may be offered in the pharmacist.’ [interview pharmacy 13].

### Problems associated with EPS2

In parallel to the advantages of EPS2 discussed, respondents described a range of problems which had occurred during the use of EPS2. These included: system failures, split prescriptions, missing prescriptions, codes not being recognized, problems with claiming, problems with nominations, problems concerning smart cards, large amount of printing, mixed ways of working, tensions with GPs and tensions with patients.

#### System failures

Twelve of the thirteen respondents interviewed reported experiencing problems when the system went down, although interview pharmacy data demonstrated that this was a bigger problem for some respondents than others. The remaining respondent had not yet received any EPS2 prescriptions. Two respondents expressed the view that the problems had become less over time. It became apparent from the analysis that there were many technological components to the system and a problem occurring in any of these components would create difficulty. These components included the internet provider, the GP practice software, the Spine itself and the message provider that transferred messages between the Spine and the pharmacy. Interview data demonstrated that the way of dealing with these difficulties was to ask the patient to come back when the problem had been resolved or offer delivery. Where more urgent acute prescriptions were needed or the system was down for a prolonged period of time, the pharmacy asked the GP practice to send the prescriptions manually. However, one respondent said they had turned EPS2 off completely and were in the process of changing message provider, after which they would turn it on again.

‘When we first started out after about 4 or 5 months. EPT2[EPS2], for some reason it has … the message transmitter or whatever they called, failed to work for 3 days, and because it was a start up Connecting to Health didn’t realise, now it took us 3 weeks nearly to sort of out 3 days of problems. Trying to get things reprinted, resorted and make sure that we had not lost money or we had got everything out. We had the same problem in June. EPT2 went down for 3 or 4 batches and it has taken somebody, a senior member of staff, two and a half days to sort it out and we just can’t be bothered any more’. [interview pharmacy 5].

‘Em. [It has gone down] a couple of times but it hasn’t really given us any problems, so if it has gone down at our end and not the doctors, we will just ring them and say, “don’t send any more down until we let you know” and if people are going for acute they’ll send them out with a green [paper] one, and then if it goes down the doctor then they just need a quiet spell and in the worst case scenario they can fax prescriptions across if it is desperate. But it hasn’t happened often’. [interview pharmacy 6].

#### Split prescriptions

Where prescriptions were split this was reported to be due to controlled drugs or drug codes not being recognised electronically in the majority of cases. However, one respondent stated that the split between electronic and manual items could be quite random. Interview data demonstrated that splitting of prescriptions caused problems, 1) because patients would arrive at the pharmacy expecting all items to be there and no one would have collected their paper prescriptions from the GP Practice (many English pharmacies operate prescription collection services for paper prescriptions), 2) because paper items take longer to process so not all items would arrive at the same time or 3) because the two sets of dispensed items were not necessarily stored together at the pharmacy. Strategies reported to have been developed to deal with these situations included pharmacy staff putting a note on the patient’s medication record to alert the pharmacist that there were items on the record that could not be sent electronically, checking the record carefully, and educating patients that some items would take longer to arrive than others. One respondent suggested that it would be helpful to have an electronic message, sent with the other items that the patient had ordered, stating that there was also a controlled drug which was being sent manually.

‘We have also done that for patients who receive any controlled drugs on a monthly basis, so we have made a note to say when you get release two prescriptions to check if there are any control drug prescriptions waiting in the surgery on paper’. [interview pharmacy 13].

‘Just I have been able to explain to patients that if they have CDs [controlled drugs] on their prescriptions, it takes, it might take longer’. [interview pharmacy 1].

#### Missing prescriptions

Five respondents reported some prescriptions had gone missing, which was related to them being sent when there were problems with the system being down in some cases. However, interview data demonstrated that missing prescriptions had not been a major problem in the majority of cases. A method used by pharmacists to prevent missing prescriptions was to not try downloading any prescriptions when the system was down, but this could only work if the pharmacist was aware the system was down. One respondent reported that missing prescriptions became a more major problem when prescriptions went missing and the pharmacist did not know that there had been an attempt to send them. The solution to missing prescriptions reported by interviewees was to ask the GP practice to reissue patients with paper prescriptions.

‘I can only think of one time when it did occur and that was because there had been an EMIS [general practice computer system]update, and the prescriptions had been done and been sent to the Spine, but they had got stuck on the Spine and there was no way of them coming down, but it affected very few people cause EMIS were aware of the problem and it got resolved within a day. The surgery reissued the affected patients with paper prescriptions’. [interview pharmacy 13].

‘We had a few lost prescriptions that we did not find out about until 4 or 5 days afterwards. It is a massive problem….Say if it is out for a couple of minutes and you don’t know, and you just happen to try and download scripts at that instant, it can actually block it’. [interview pharmacy 8].

Five respondents also reported that prescriptions sometimes got delayed on the Spine, which could be a particular problem with acute prescriptions and where the GP Practice and pharmacy were in close proximity. However, this was not a problem experienced by all pharmacies.

‘What happens if they need medication, they have seen the doctor, because the surgery is so close to us, and the doctor says, ‘I have sent it to the pharmacy’ and then they [the patients] arrive here but the prescription isn’t here. And depending on the electronic traffic I get that goes through the system, if it is too busy then it takes even longer to arrive’. [interview pharmacy 2].

‘No, well even if we get acutes when they have been in to see the doctor that morning, they are usually here within about 5 minutes’. [interview pharmacy 13].

#### Codes not being recognised

Problems reported with codes not being recognised applied to both medication and dosing instructions. Some respondents reported that these problems were reduced over time. Others stated that they were resolvable if the GP Practice or pharmacy staff knew what to do. Another reported that he had to convert the code manually each time.

‘As time goes on the drugs are available’. [interview pharmacy 7].

‘When ETP started, P my second pharmacist had to go next door and put most of the stuff in a useable form’. [interview pharmacy 5].

‘It processes it their end, but obviously whether it is in the DM + D dictionary or not, it makes a difference, so sometimes at this end we have to pick it up manually and say, ‘these two are the same’ and try and do it that way’. [interview pharmacy 12].

#### Problems with claiming

Difficulties reported with claiming for dispensing included the cumbersome nature of carrying out prescription endorsements and concern that money was being lost on some items. Two respondents stated that no accurate report was automatically generated of how many items had been submitted for payment so the pharmacist had to count them manually to check they had been paid the correct amount. Other cumbersome features reported were having to change screens to endorse items (one respondent) and having to go through everything line by line to check if any extra endorsing was needed (one respondent). Other reported problems were that a few items did not endorse automatically ( two respondents) and that none of the items on a prescription could be claimed for until any owings were cleared (one respondent). One of the respondents stated that the number of items not endorsed automatically had reduced in time and that his software provider was able to assist where necessary and the other said he had learned how to resolve them. Difficulties were also described where items were not in the drug tariff, were ‘specials’ or required NCSO [no cheaper stock obtainable] endorsement because items were not available at the usual price (five respondents). Problems were reported to occur when claiming for these items because any mistakes made could not later be rectified and because the NCSO price had not always been published at the time of dispensing. One respondent described dealing with this problem by asking the GP Practice to issue items requiring ‘special’ endorsing on paper prescriptions. Whilst the majority of respondents reported difficulties with claiming, two respondents explicitly stated that they had not experienced any problems, although one of these respondents was not the owner and did not directly deal with the financial side of the business.

‘I mean, good very accurate, I mean, except for specials; I don’t think that it lends itself to items that are not in the drug tariff’. [interview pharmacy 15].

‘It is very straightforward and all the information that you need to record is on each section and so for one of the specials, you can go into that and it brings up all the information up that you need to include’. [interview pharmacy 13].

#### Problems with nominations

Respondents reported a range of problems with nominations and different issues were raised by different respondents. These included: patients being nominated to a different pharmacy without their consent (three respondents experienced and one respondent concerned), GPs nominating the wrong pharmacy (two respondents), GPs telling the patient to collect their prescription from the closest pharmacy without checking that that was the one they were nominated for (two respondents), the system not allowing a particular patient to be nominated (one respondent), patients being denominated from electronic prescribing without their knowledge (one respondent), and nomination not being changed back after having been changed to allow access to out of hours services (one respondent). One respondent stated that the problem with GPs nominating the wrong pharmacy occurred because s/he had selected the wrong branch of a chain. Interview data demonstrated that problems with nominations were resolved by pharmacists changing the nomination back to the correct one and patient education.

However, problems with nominations were not experienced by all interviewees. Two respondents reported that patients came to the wrong pharmacy but that the problem had not increased since EPS2 implementation as the same thing could happen with paper prescriptions when pharmacies offered prescription collection services and patients did not remember which pharmacy they had asked to pick up their prescription. A further two respondents had experienced no problems with nominations.

‘We have found that quite often that doctors have nominated the wrong pharmacy when they have had the patients sat in front of them. And the other thing is sometimes the GP, and we will have had this a few times, where they have sent the patient over the road to pick up the prescription from us, not realising that the patient has been nominated at a different pharmacy. And so we have been going ‘sorry it has taken a little while to come, its taking a little while to come’. We said, ‘has the doctor just nominated?’ ‘No I use [name of pharmacy] and you say, ‘sorry that is where you will get your prescription’. ‘The doctor said ‘go straight over the road’”. That is obviously not the system’s fault, it is a training issue with the GP’. [interview pharmacy 14].

We had some trouble; patients were nominated allegedly by a different pharmacy without their consent. There was a problem that without their consent that someone else nominated them while they were expecting the prescription to come here on paper. It went to a different pharmacy electronically [interview pharmacy 9].

‘That is not the fault of the EPS2. The same thing happens with a lot of them with their paper prescriptions. It tends to be the ones that tend to go between pharmacies that have the problems [interview pharmacy 8]’.

#### Problems concerning smart cards

Some issues were reported with smart card use [cards to permit user log in] but it came apparent from the interview data that these had not caused major problems. Two respondents reported that there had been some issues with smart cards not working. The smart card system was set up so that each individual would use their own smart card. However, two respondents identified that there was some flexibility with smart card use. There were mixed reports about smart card availability for locums, with two respondents stating that their locums did have smart cards and three stating that they were not available. However, this was not considered to cause major difficulty as smart cards were only needed to download and send off prescriptions, and they could be downloaded by a dispenser. One respondent reported experiencing a problem with an expired smart card at the GP Practice end which meant that the prescription could not be downloaded by the pharmacist.

‘We only have two in the dispensary and people have left and they have not been replaced, and one of them is out of date and doesn’t work. I mean, what tends to happen is that one smart card gets put in and everyone uses the smart card. …So it doesn’t actually kind of lend itself to everyone doing their own work on their own smart card’. [interview pharmacy 15].

‘Actually you don’t need a smart card all the time. Once you have it downloaded you can do quite a lot without a smart card even in’. [interview pharmacy 6].

#### Large amount of printing

Five respondents raised the issue of the large amount of printing which the pharmacist has to do with EPS2. Respondents expressed the view that whilst in theory, EPS2 was meant to be a paperless system, this was not borne out in practice and the printing had instead been transferred from the GP Practice to the pharmacy. Printing of tokens was reported to be necessary because pharmacists needed something to dispense and check against and could not keep the computer screen showing one prescription while these processes took place. In addition, respondents reported having to print out exemption declaration forms for patients and repeat forms. One respondent complained that the repeat forms automatically printed out in duplicate where there were more than four items on the prescription creating even more paperwork. Respondents expressed the view that this caused expense to the pharmacists which were not met by the allowance given for using EPS2.

‘I’m paying for the tokens, the NHS … isn’t. The NHS isn’t paying for the green forms, because they are not going to be using them. They are going to be emailing, so there is not going to be a paper … I’ve got to pay for printing out. I’ve got to … pay for the toner and I have to pay for the paper. I think that somebody somewhere has thought that is a good jape. We will get the pharmacists to pay for it’. [interview pharmacy 11].

‘If you think about the demography of prescriptions, most of them are people with exemptions and most of those people will order their own medication, so they need a reorder slip. You have to print off a token, and if you are any way busy you can’t be running around checking screens. You have to have something paper to check. If you have got to do MURs and this that and the other, you really have to. Sometimes, when you are doing repeats, the script has to wait to be checked. You can’t keep a screen on hold, while someone finds the time to come and check it’. [interview pharmacy 5].

#### Mixed ways of working

Two respondents were of the view that it would be easier if they received all prescriptions electronically rather than some electronically and some manually. In one case, the GP Practice sometimes sent them electronically and sometimes manually and in the other case, one of the two GP Practices attached to the pharmacy had gone live and the other hadn’t.

‘Well, like when all our surgeries go to the same system, it will just make it easier for us to be working on. Any one system is easier to deal with rather than a number of systems’. [interview pharmacy 6].

Whilst the majority of respondents expressed the view that EPS2 reduced error, two respondents expressed concerns relating to safety and security. One respondent was of the view that manually endorsing prescriptions could act as an extra checking stage and that automatic endorsing took that away. The second expressed the opinion that electronic transfer of prescriptions may be insecure as patient’s records could get hacked into or an individual could fraudulently claim to be a particular patient in order to obtain their medication.

‘People’s telephones can get hacked into … and probably it can with somebody determined. I don’t know if anybody has tried it.… The other thing is of course okay the doctor is not going … to be issuing paper to the patient. How do you necessarily know for example if it isn’t a regular patient, and they have said, ‘I want my prescription at so and so pharmacy, I passed it on my way into the surgery, I want it sent there’. They know nothing about the patient. Are they going to be given, some sort of paper collection data, something in their hand to say that they are going to be collected a prescription with a code number or something on it. I am not sure about that’. [potential problem interview pharmacy 11].

#### Difficulties with supplementary prescribing

Two respondents discussed a problem they had experienced accessing prescriptions written by nurse prescribers. This was reported to be due to the fact the nurse had selected the GP rather than nurse prescription form. One of the respondents started that this problem was exacerbated because the system allowed the prescription concerned to be sent at the GP Practice end but that this could not then be downloaded at the pharmacy end. A suggestion was made that nurse prescribers should be alerted by the software at their end that the prescription was being blocked so that the problem could be resolved speedily at source.

#### Time consuming

Two respondents expressed the view EPS2 was time consuming for the pharmacy because of claiming (see above) and getting patient consent for nominations.

‘It saves the surgery time; the government save time and the pricing bureau, the only people who have more time to work on it are pharmacists’. [interview pharmacy 5].

#### Tensions with GP Practices

It became apparent from the analysis that whilst EPS2 provided opportunities to work closely with GP Practices, it also created some tension. Six respondents reported experiencing some difficulties although in the majority of cases respondents were of the view that this had not caused a large decline in overall relationships. Interview data demonstrated that tensions occurred where the EPS2 process had failed or where there were general failures in communication.

Interview data demonstrated that there had been some difficulties in initialising EPS2 at the GP Practices. Four respondents reported that they had had problems with GPs not sending prescriptions electronically. Two of the respondents stated that they had not yet received any EPS2 prescriptions, one that they had received them from some but not all GP Practices and one that they had received few to begin with but that had now been resolved. A fifth respondent expressed the view that there was an enormous amount of work involved for GP Practices in setting up EPS2.

‘Initially we did have a problem, but now it is not a problem, they have all realised that for it to work, we need to work together. …. I would have thought that it should have improved it over all, but obviously with certain individuals it can get fraught because they don’t see the error of their way because, historically GPs are just used to saying to pharmacists, ‘we are right and you are wrong’. ‘[interview pharmacy 12].

‘We know that they are live, they are going live, the PCT has told that they are live, but we are just having some problems getting them to send us prescriptions electronically [interview pharmacy 10].

‘I thought yeah, we will switch it over; there will be a few problems. I was surprised how much more they’ve had to alter. It has been a fairly mammoth thing … at the GP’s end’ [interview pharmacy 8].

#### Tensions with patients

Five respondents described tensions that could occur between the pharmacy and patient as a result of EPS2. It became apparent from the analysis that these tensions were caused either by patients becoming annoyed when the system did not work as it should or by patients having unrealistic expectations of what the system could do even when functioning correctly. Respondents reported that not all patients realised that they still had to actually order their prescriptions each month. Another cause of patient irritation reported was the expectation that the prescription would be ready for patients when they get to the pharmacy. In reality the prescription may take a few minutes to arrive (as above) and then has to go into the dispensing queue with paper prescriptions which other patients have brought in. Interview data demonstrated that in the majority of cases, these tensions could be resolved by educating patients and GP Practices so that patients’ expectations were more realistic. However, in one pharmacy, patient irritation was reported to have been one of the factors leading to the discontinuation of EPS2. Another respondent was concerned that problems with the system at these early stages made it more difficult to “sell” the service to patients.

‘We try to explain to them that the only thing that is going to change is that it is going to come electronically and always to the same pharmacy, rather than getting lost in the system or kind of like just sitting on the bench in the surgery’. [interview pharmacy 2].

‘Plus the irate patients is one of the main reasons as well to switch it off. We are tired of being called “idiots”. It is not our fault’. [interview pharmacy 5].

#### Other problems

There were a range of problems only raised by individual respondents. Some of these were anticipated problems from a pharmacist who had not yet received any EPS2 prescriptions and included: concern that she would no longer be able to obtain seven day prescriptions for patients using monitored dosing systems, apprehension that if the pharmacist had a query with a downloaded prescription it may be more difficult to leave it half done on the screen while it was resolved, and concern that problems would occur with the management of centralised dispensing i.e. where the branch in which prescriptions were dispensed was not the same as that from which prescriptions were collected. Problems raised by an interviewee who was on the implementation board for EPS2 as well as being a practicing pharmacist included the repeat forms printed off by the pharmacist being in seemingly random rather than alphabetical order and messages to patients (for example, flu vaccination due) coming together with repeat forms so that they may be less likely to be noticed by pharmacists and passed on to patients than if they were separate. A third respondent raised the issue of repeats coming too early, but was of the view that this had been more or less resolved. The same respondent also discussed the fact that when there were breakdowns with the EPS2 system for prolonged periods this could worsen workflow rather than improve it because there may be one day where no prescriptions can be dispensed and then the next double the amount would need to be done. A fourth respondent reported that she had experienced some difficulties when patients wanted their repeat dispensing prescriptions early if, for example, they were going on holiday. She discussed finding a solution to this by printing out the token and scanning it back in, which then allowed the repeat to come through earlier. Finally, a fifth respondent described the difficulties the GP Practice had experienced in getting their staff trained as they did not have funding to pay their staff to stay out of surgery hours for training, and during surgeries the training was constantly being interrupted.

### Further suggested improvements

The majority of interviewees had some further suggestions of how EPS2 could be improved. After discussing problems and concerns, interviewees were asked if they had any further suggested improvements for EPS2. Nine of the eleven respondents had suggestions and these varied between respondents. Some of the suggestions related to GP Practices, others to the technology and one to patient education.

#### Suggestions relating to GP Practices

Two respondents suggested that further training for the GPs on EPS2 would be helpful and another was of the view that it would be helpful if pharmacists were able to send messages to GPs; at present the system only allowed one way communication between the GP Practice and the pharmacy. Another respondent reported that increased pressure from the PCT for General Practices to use EPS2 could be helpful. Yet another was of the view it would be helpful for the pharmacy to be involved when the software for EPS2 was being installed at the GP Practice so that there was a shared understanding of what was involved.

“Something you can include in your thing is that when they implement systems in GP Practices, they should look to invite their local pharmacist there as part of the training” [interview pharmacy 12].

#### Suggestions relating to technology

Other interviewees raised suggestions concerning the technology itself. These included: 1) developing a portable device for EPS2 that the pharmacist could use when checking the prescription rather than having to print the prescriptions, 2) improvements in the software automatically translating GP directions into suitable directions for the patient, 3) having an automatic message come up on the Patient Medication Record to indicate whether the prescription had been sent electronically or manually (making it easier to trace when patients came in) and 4) allowing repeat dispensing prescriptions to be downloaded earlier if necessary. Finally, one respondent was of the view that there should be an electronic means for sending feedback messages to the various parties involved with EPS2.

‘But also if they could bring in a mobile device so you have a screen to check against, then there will be a huge benefit paper wise’ [interview pharmacy 6].

‘Connecting for Health should have insisted on something so that the system suppliers when they created the software that they would have had that inbuilt into it that this is a system we use for communicating with us, or with the Connecting for Health with the PCT, or you know because if you have got a nice all singing and all dancing system, why not use it to send messages’. [interview pharmacy 12].

#### Suggestion relating to patients

Another suggested improvement was education of patients that this was a change for the NHS as a whole, rather than individual pharmacies trying to get more prescriptions.

### Feedback procedures and assistance with problems

A majority of respondents (eight) reported that assistance was available when problems arose with EPS2, but a majority (eight - but not the identical eight) expressed a level of dissatisfaction with the support available. Sources of assistance reported included the software provider, Connecting for Health and the PCT. A range of problems were described with the support available including it taking too long to be put through to the correct people to help, feedback not getting through to the right people, office people not understanding community pharmacy in practice, problems taking too long to be resolved and not being informed when a problem had been resolved. One respondent described the frustration he experienced when trying to get assistance and it became apparent that this was due to the system comprising many components and no one taking overall responsibility. Some respondents who had been part of the initial pilot scheme described changes to feedback procedures over the course of time. Two respondents were of the view that as more sites had gone live, less support was available as there were more people needing support and software companies were less interested in feedback than at the initial stages. Conversely, another respondent reported having experienced more frustration at the beginning as fewer support staff knew about EPS2 and it had taken time to get through to the right people. One respondent expressed the concern that changes to the NHS would further reduce the level of support available. Finally, one respondent reported that he had not tried to use any feedback mechanisms and did not have contact details of any particular person to contact if he experienced problems.

‘The time that it happened, as I said, I have been able to get through to the help desk and the help desk has been able to solve it for me’. [interview pharmacy 1].

‘But it is so cumbersome, you have to pick about 15 different options before you get right down to ‘can you please call me’. [interview pharmacy 12].

‘But anyhow, I think that probably if he can get to the right people then the feedback is taken seriously and if they don’t it is probably just ignored’. [interview pharmacy 14].

‘I think that the other issue that is going to happen is when the PCTs disappear, I think that there is going to be no support for anyone’. [interview pharmacy 12].

## Discussion

It became apparent from our analysis that respondents perceived a wide range of advantages of EPS2 including improved safety, stock control, time management and doctor-pharmacy relationships. However, these did not match the potential advantages originally suggested by the Department of Health and NHS Connecting for Health [1-3] which included reduction of illegible prescriptions, promotion of repeat dispensing and reduced effort in prescription reimbursement. Although pharmacists reported an increase in repeat dispensing, only one expressed the view that this was one of the benefits of EPS2. In addition, respondents did not perceive a large difference in terms of work processes or development of their professional role. A sample of Canadian pharmacists interviewed by Motulsky et al. [10] identified more perceived change in terms of their professional role. However, in the Canadian system being evaluated, clinical information was given to patients; in the UK at present pharmacists do not receive any additional information over that received using the paper based system.

A large number of problems had been experienced in relation to both the technology itself and the way it was used by General Practices. Difficulties reported by the majority of respondents included the system going down, problems resulting from prescriptions being split with some items coming electronically and some manually, problems with codes not being recognised, problems with claiming reimbursement and problems with nominations. It became apparent that work-around procedures had been developed for dealing with these issues but that not all these problems had been addressed sufficiently at source. This sometimes had implications for the extent of EPS2 use and also limited some of the potential advantages of the EPS2 service, such as reduced effort in the management of prescription reimbursement. Respondents made suggestions for future improvements to EPS2.

While interview data demonstrated that there were some feedback procedures in place, these were not regarded as being sufficient by the majority of respondents. Difficulties experienced included it taking too long to be put through to the correct people to help, feedback not getting through to the right people, office people not understanding community pharmacy in practice, problems taking too long to be resolved and not being informed when a problem had been resolved.

The findings support and build upon those found by very early interview work including: improvements to workflow with EPS2, problems with missing prescriptions and an overall relatively positive attitude to EPS2 despite problems experienced. However this study has identified many other issues that have arisen as the system has matured further. It has also clarified that whilst workflow was improved overall, system failures could lead to a less even workflow at times. Our findings also build on exploratory work identifying interventions made by pharmacists when dispensing EPS2 prescriptions [8]. Problems which needed to be resolved included split prescriptions between paper and electronic, missing prescriptions and use of Latin abbreviations on directions which needed to be changed by the pharmacist. These problems, amongst many others, were all reported by the pharmacists in the present study.

Many of our findings are also supported by previous surveys of community pharmacy staff attitudes towards electronic prescriptions in America and Sweden [11,12]. Positive features identified in both our study and at least one of the other studies [11,12] included improved speed/efficiency, reduced interruptions, increased prescription integrity/security and improved relationships with prescribers. Negative features were prescribing errors, delays in receiving the electronic prescription, technical problems, reduced communication with prescriber or patient, electronic prescriptions being lost or sent to the wrong pharmacy, no alert in computer that an electronic prescription has been received, decreased prescription integrity/security, that prescribers used different medication codes to pharmacists and that electronic prescriptions could not be used for controlled drugs.

However, there were some differences in the findings between our study and others. Legibility of prescriptions was a benefit identified from the American and Swedish surveys of electronic prescriptions but not from our analysis of the English EPS2 service. Also, Rupp et al. [11] identified missing or insufficient information on the electronic prescription as a problem; this was not identified in our study. These differences were probably due to electronic prescribing already being widely implemented in England prior to the introduction of electronic transfer. In addition, 28% of the respondents in Sweden stated that it was more inconvenient to make changes or corrections with electronic prescriptions, a problem we did not identify in our English study. The difference was probably related to differences in the particular systems used in Sweden versus England. Novel issues identified by a large number of respondents in our study that had not been identified in previous work included improved stock control, problems with split prescriptions and problems with claiming reimbursement.

### Limitations

This was an exploratory study of the experiences of pharmacists in England who were early adoptors of EPS2. The sample was fairly small and taken from PCTs who were proactive in promoting EPS2 which had the potential to introduce bias. However, we were able to obtain views from a wide range of pharmacists including those working in different types and sizes of pharmacy, in diverse locations and with a varying range of experience of EPS2. Pharmacists who had very negative views of the system may have been less likely to participate. On the other hand, expressing these views and trying to evoke change may have also been a reason for participation. A number of pharmacists did express a great deal of frustration with aspects of EPS2 during interviews and this had not deterred them from participating in the interviews.

## Conclusions

Pharmacists reported little process change as a result of the introduction of EPS2. Respondents described a large number of problems that had occurred and the majority of respondents did not find feedback procedures satisfactory in terms of resolving problems and improving the service. However, beyond all these difficulties respondents could see many advantages of the EPS2 system and perceived it as the way forward. Even the respondents who reported experiencing a great deal of frustration did not express an overall negative view.

## Abbreviations

EPS: Electronic prescription system; EPS1: Electronic prescription system release 1; EPS2: Electronic prescription system release 2; DM + D: Dictionary of medicines and device; PCT: Primary care trust.

## Competing interests

The authors declare that they have no competing interests.

## Authors’ contributions

SG conducted and analysed the interviews and wrote the paper. RH was involved in the recruitment of the pharmacists, the designing of the interview schedule and editing the paper. NB oversaw the evaluation and was involved in editing the paper. All authors read and approved the final manuscript.

## Authors’ information

SG is a postdoctoral researcher at UCL School of Pharmacy and is currently on secondment to the Centre for Medication Safety and Service Quality (CMSSQ), Imperial College NHS Healthcare Trust. Her research interests include adherence, concordance and medicines safety. RH was a research fellow with UCL School of Pharmacy at the time this research was conducted and worked on the Evaluation of the Electronic Prescription Service in Primary Care. NB is Professor in the Department of Practice and Policy at UCL School of Pharmacy and a Visiting Professor in Patient Safety at Harvard Medical School. He is Director of Research and Education at The Health Foundation.

## Pre-publication history

The pre-publication history for this paper can be accessed here:

http://www.biomedcentral.com/1472-6963/13/435/prepub

## Supplementary Material

Additional file 1 Interview Schedule for pharmacists. Click here for file
