# Supplementary material for: English community pharmacists’ experiences of using electronic transmission of prescriptions: a qualitative study
Source: BMC Health Serv Res. 2013 Oct 23;13:435. doi: 10.1186/1472-6963-13-435 (PMC4015561; doi:10.1186/1472-6963-13-435)
Supplement: Additional file 1 — Interview Schedule for pharmacists. [file 1472-6963-13-435-S1.rtf]

Additional file 1 Interview Schedule for pharmacists
INTRODUCTION TO THE INTERVIEW
Hi.  I'm Sara Garfield.  I'm a pharmacist from The School of Pharmacy, UCL.  Thank-you for your time.  We just wanted to take a few minutes to find out your experiences with the EPS system.   We are evaluating the service as a whole and looking at how it could be developed further and any further support pharmacists might need.  All information will be treated confidentially. Our report will not contain information about individual pharmacies.

CANDIDATE INTERVIEW ITEMS
1	Adoption of the EPS System
Why did your pharmacy decide to adopt the EPS system?
What training did you receive to help you with adoption of EPS?
Prompts on the quality, timing and relevance of training.

2	Use of the EPS System
Which types of prescriptions does the pharmacy receive electronically?
Prompts on whether the site receives  acute, repeat prescribing and/or repeat dispensing prescriptions.

If repeat dispensing prescriptions have been used
Do you/have you in the past used paper repeat dispensing?
How do you find using the EPS system?

 How has the process of dispensing changed following the introduction of EPS?
Prompts: on whether there has been creation of new standard operating procedures by pharmacist or company changes to repeat dispensing processes if relevant

What are the benefits of EPS?
Prompts: on whether there have been effects on workflow, owings or improved accuracy of Patient Medication Records
	
What concerns have you had with EPS?
Prompts on security

What problems have you encountered with EPS?
Prompts include whether there have been missing prescriptions, prescriptions arriving late, prescriptions split between batches, prescriptions split between paper and electronic forms –especially monthly repeat prescriptions, whether the system has been down, whether there have been missing smartcards, whether there have been any problems emerging from the prescribing of controlled drugs, whether there have been any changes to the availability of dispensing repeats due to waiting for NCSO endorsements, whether there has mismatching of coding between prescribing and dispensing soft ware causing problems, whether there have been problems with dispensing different flavours, whether there are a lack of prompts when the Patient Medication Record is updated.

How have these problems and concerns been addressed?

Are there any feedback procedures that you can use?
 Prompts: to software companies

3	Changes Emerging from Use of the Electronic Prescription Service
What effects do you think EPS has on ease of accurate dispensing?
Prompts on whether there are changes to workflow, use of barcodes and transcription, delayed items.	

How has the introduction of EPS affected pharmacists' professional role?

How has the introduction of EPS affected the general relationships between general practices and pharmacies?
Prompts on whether prescriptions are received from a wider/ narrower range of surgeries, whether there are  tensions when problems occur such as missing/delayed prescriptions, and whether there are changes in the handling of nominations

How could EPS be improved for your pharmacy?
Prompts on whether changes in technology, GP/pharmacy relationships, or work practices are required.

4	Information about the Community Pharmacy
Finally a few quick questions about the pharmacy
What computer system do you use for dispensing ?
How many pharmacists work at the site?
How many pharmacists work at the site?
What type of pharmacy is this?

CLOSE OF THE INTERVIEW
The participant is thanked for their time and provided with an opportunity to make any other comments that he or she might wish with regard to local deployment of electronic and paper repeat dispensing prescriptions. Participants are also given the opportunity to ask any remaining questions they might have about the project.
